# Endoglin Haplo-Insufficiency Modifies the Inflammatory Response in Irradiated Mouse Hearts without Affecting Structural and Mircovascular Changes

**DOI:** 10.1371/journal.pone.0068922

**Published:** 2013-07-24

**Authors:** Ingar Seemann, Johannes A. M. te Poele, Sophia J. Luikinga, Saske Hoving, Fiona A. Stewart

**Affiliations:** Division of Biological Stress Response, The Netherlands Cancer Institute, Amsterdam, the Netherlands; Cardiovascular Research Institute Maastricht, Maastricht University, Netherlands

## Abstract

**Background:**

It is now widely recognized that radiotherapy of thoracic and chest wall tumors increases the long-term risk of cardiovascular damage although the underlying mechanisms are not fully elucidated. There is increasing evidence that microvascular damage is involved. Endoglin, an accessory receptor for TGF-β1, is highly expressed in damaged endothelial cells and may play a crucial role in cell proliferation and revascularization of damaged heart tissue. We have therefore specifically examined the role of endoglin in microvascular damage and repair in the irradiated heart.

**Materials & Methods:**

A single dose of 16 Gy was delivered to the heart of adult Eng^+/+^ or Eng^+/−^ mice and damage was evaluated at 4, 20 and 40 weeks, relative to age-matched controls. Gated single photon emission computed tomography (gSPECT) was used to measure cardiac geometry and function, and related to histo-morphology, microvascular damage (detected using immuno- and enzyme-histochemistry) and gene expression (detected by microarray and real time PCR).

**Results:**

Genes categorized according to known inflammatory and immunological related disease were less prominently regulated in irradiated Eng^+/−^ mice compared to Eng^+/+^ littermates. Fibrosis related genes, TGF-β1, ALK 5 and PDGF, were only upregulated in Eng^+/+^ mice during the early phase of radiation-induced cardiac damage (4 weeks). In addition, only the Eng^+/+^ mice showed significant upregulation of collagen deposition in the early fibrotic phase (20 weeks) after irradiation. Despite these differences in gene expression, there was no reduction in inflammatory invasion (CD45+cells) of irradiated Eng^+/−^ hearts. Microvascular damage (microvascular density, alkaline phosphatase and von-Willebrand-Factor expression) was also similar in both strains.

**Conclusion:**

Eng^+/−^ mice displayed impaired early inflammatory and fibrotic responses to high dose irradiation compared to Eng^+/+^ littermates. This did not result in significant differences in microvascular damage or cardiac function between the strains.

## Introduction

Nearly 5 million long-term cancer survivors were registered in 2007 in the United States alone and at least half of these patients underwent radiotherapy as part of their cancer treatment. Although radiotherapy is an effective cancer treatment, it can contribute to late toxicity in surrounded normal tissue. Much work has been done to reduce the risk of late normal tissue toxicity induced by radiotherapy over the last decade, including modified fractionation schedules and conformal image-guided-radiotherapy (IGRT). Moreover, knowledge about the molecular mechanisms underlying the development of normal tissue toxicity after radiotherapy is increasing and this should eventually help in designing methods to prevent or treat normal tissue toxicity. Nevertheless, little is known of the underlying molecular mechanism of radiation-induced cardiac toxicity in thoracic cancer patients.

We and others [1], [2], [3] previously demonstrated that high dose cardiac irradiation induces microvascular damage and capillary loss, eventually leading to fibrosis. Radiation-induced fibrosis, defined by excessive fibroblast proliferation, myofibroblast differentiation and overproduction of extracellular matrix, is predominantly induced by activated Transforming Growth Factor-β1 (TGF-β1) [4]. TGF-β1 has been defined as the master switch in the fibrotic program and it acts on at least three different biological activities: regulation and inhibition of cell growth; immunosuppressive activities; and regulation of extracellular matrix component deposition [5]. Numerous studies have shown correlations between increased severity in radiation-induced normal tissue toxicity and TGF-β1 signal activation [6], [7], [8]. For regulation of endothelial function by TGF-β1, signaling of type I receptors ALK1 and ALK5 are the most important. Regulated (R-)Smads, phosphorylated by type I receptors, form heteromeric complexes and these accumulate in the nucleus where they regulate the transcription of specific target genes [9]. Endoglin, a co-receptor for TGF-β1, is highly expressed in proliferating endothelial cells and plays a crucial role in angiogenesis. Since endoglin has no kinase domain itself, it promotes TGF-β1 signaling through ALK1 receptor to promote cell proliferation and migration [10], [11], [12]. Mice that are deficient in endoglin die in mid-gestation due to vascular and cardiovascular defects. Moreover, mice carrying a single copy of the endoglin gene show a tendency to develop hereditary hemorrhagic telangiectasia (HHT) phenotype as they age, with extensive dilated and weak-walled vessels [13], [14]. Disease prevalence depends on the genetic background of the mice (7% in C57Bl/6 and 72% in Ola mice age 1 year). This phenotype is similar to radiation-induced microvascular damage, which raises the question of whether endoglin may also play a crucial role in radiation-induced cardiac injury. To explore this, we used a model of radiation-induced cardiac injury in Eng^+/−^ mice and compared this to damage in wild type littermates.

## Methods

### Mice and irradiation procedure

Eng^+/−^ C57BL/6 mice were originally obtained from H. Arthur (Institute of Human genetics, International Centre for Life, Newcastle upon Tyne, UK) and subsequently bred in the Netherlands Cancer Institute. Male Eng^+/+^ mice and Eng^+/−^ littermates aged 8–12 weeks were randomly allocated (after genotyping by PCR) to receive 16 Gy or 0 Gy to the heart. Mice were housed in a temperature-controlled room with 12 hour light-dark cycle. Standard mouse chow and water were provided ad libitum. Irradiation was performed with 250 kV X-rays, operating at 12 mA and filtered with 0.6 mm Copper. The dose rate was 0.94 Gy/min with a field size of 10.6x 15 mm (including up to 30% lung volume) and the rest of the mouse was shielded with lead. Unanesthetized mice were immobilized in a prone position in acrylic perspex jigs. Separate cohorts of animals were included for analyses at 4, 20 and 40 weeks after irradiation, with age matched controls (sham irradiated with 0 Gy). Each cohort typically comprised 10 to 15 mice (n = 130 in total). This study was in agreement with the Dutch law on animal experiments and welfare, whereby the Animal Experiments Committee (AEC) of the Netherlands Cancer Institute has evaluated the set-up of the experiments and has given a positive recommendation (Permit number: 08008–1990), and in line with the international *Guide for the Care and Use of Laboratory Animals* (Eighth edition). No severe suffering was anticipated in this study. If mice appeared distressed, or lost >15% body weight, they were humanely sacrificed before the planned follow-up time. At termination of the experiment, mice were humanely sacrificed under lethal sodium pentobarbital anesthesia (18 mg per mouse, i.p).

### Gene expression profiling and pathway analysis

Total RNA was isolated from frozen sections (30 sections of 30 µm per mouse and 4–7 mice per group) of the mid part of the heart using Trizol® Reagent (Invitrogen Corporation, Carlsbad, USA), according to the manufacturer’s protocol. The quantity of total RNA was measured using a spectrophotometer (NanoDrop, Thermo scientific, Wilmington, USA) followed by a quality check measured by a Agilent 2100 Bioanalyzer with the RNA Integrity Number (RIN) (Agilent technologies, Santa Clara, USA). Samples with a RIN above 7 were used for DNAse treatment and amplified (350 ng per sample) using Illumina Totalprep RNA Amplification kit (Ambion, Grand Island, USA). Before hybridization, individual RNA was pooled for each treatment group. Hybridization of aRNA to Illumina Expression Bead Chips Mouse Whole Genome (WG-6 vs. 2.0) and subsequent washing, blocking and detecting were performed according to the manufacturer’s protocol (Illumina, San Diego, USA). Samples were scanned on the IlluminaR BeadArray™ 500GX Reader using IlluminaR BeadScan image data acquisition software (version 2.3.0.13). MouseWG-6 vs. 2.0 BeadChip contains the full set of MouseRef-8 BeadChip probes with additional 11,603 probes from RIKEN FANTOM2, NCBI REfSeq as well from the MEEBO database.

Before analyzing, the database was normalized using robust spline normalization method within the microarray facility of the Netherlands Cancer Institute. Log2 ratio between expression of genes from control mice and expression of genes from irradiated mice were calculated using Excel version 2003, as well as the sum of the expression of genes from both control and irradiated mice. According to the sum of both expressions, genes with sums below 6 were discarded. The threshold for standard deviation (SD) was set to 3 and mean ± nSD was calculated to identify genes that are above expression 6 and above threshold 3 of SD. These genes were further analyzed in Ingenuity Pathway Analysis (IPA) version September 2011 core analysis. IPA calculates a significant score for each associated network. This score indicates the likelihood that the assembly of a set of focus genes in a network could be explained by random chance alone. Networks with scores of 2 or higher have at least a 99% confidence of not being generated by random chance alone. For individual gene expression profiling, RNAs of each treatment group were individually transversely transcribed into cDNA. Expression of genes of interest was detected by qPCR with SYBR Green (Applied Biosystems, Carlsbad, USA). Changes in gene expression were analyzed with the comparative ΔCt method and corrected for the expression of the housekeeping gene GAPDH. Primers used to detect changes in gene expression are listed in the Table S1.

### Tissue preparation for histology

At termination of the experiment, the heart was perfused via the aortic arch (retro-grade), under lethal sodium pentobarbital anesthesia (18 mg per mouse, i.p), with PBS (frozen sections) or PBS followed by 1% paraformaldehyde (paraffin sections). The heart was then quickly excised before freezing on dry ice or immersion in 1% paraformaldehyde.

Cross-sections were cut at the level of the mid-horizontal plane of the heart from fixed paraffin-embedded tissues (4 µm) or frozen tissues (7 µm).

#### Paraffin sections

Transverse sections were stained with hematoxylin and eosin (H&E) to measure the epicardial and myocardial thickness. To determine the extent of inflammation, sections were immuno-labeled with anti-CD45 antibody (1:400, Becton&Dickinson, Franklin lakes, USA). Perls’-staining was performed as indicator of previous hemorrhage. Based on a Sirius red staining, interstitial collagen was determined in the subendocardium and myocardium of the left ventricle (LV). Within one follow-up time all sections were processed identically, at the same time with precisely the same incubation times for the primary and secondary antibody and diamminobenzidine (DAB) solution (Sigma, Zwijndrecht, the Netherlands). Therefore, all differences between the treatments are ultimately due to DAB identification of the relevant protein.

Photographs of the LV wall, excluding the septum, were taken using a 5x objective (Leica DFC320) and 12 measurements per heart were performed for the epicardial and myocardial thickness. The number of CD45+ cells per section was counted separately in the epicard and myocard to determine the extent of inflammation. Perls’ stained sections were examined for evidence of iron-containing macrophages and this was recorded as positive or negative for each section. Interstitial collagen was quantified in five randomly selected areas of the subendocardium and myocardium of the LV (40x objective) and results were expressed as percentage tissue positive for Sirius red relative to myocardial area. Morphometric parameters were analyzed using a computerized morphometry system (Leica Qwin V3, Leica, Rijswijk, the Netherlands).

#### Frozen sections

An anti-CD31 antibody (1:50, Becton&Dickinson) was used to visualize cardiac vasculature of the central part of the heart. To determine functional changes in the microvasculature, a histochemical staining with Naphtol AS-MX/DMF and fast Blue BB salt was performed to detect endothelial cell alkaline phosphatase. Sections were also reacted with antibodies against von Willebrand Factor (vWF) (1:4000, Abcam, Cambridge, USA) as a marker of thrombotic changes. Within one time group all sections were processed identically, at the same time with precisely the same incubation times for the primary and secondary antibody and DAB solution. A double staining was performed to visualize the vasculature (anti-CD31) and the pericytes coverage (anti-NG2, 1:200, Chemicon, Temecula, CA). Primary antibodies were visualized with Alexa Fluor (AF) 633 (1:100, Invitrogen, Carlsbad, CA) and AF568 (1:250, Invitrogen).

For quantification of microvessels, five random fields (40x objective) from transverse sections of the subendocardium were photographed with a CCD 2 – Color Microscope system, including a Zeiss AxioCam color camera (Axiocam HRc, Zeiss, Göttingen, Germany), and a computerized morphometry system (Leica Qwin V3) was used to quantify the microvascular density (MVD). Vessels beneath a size of 1.5 or above 200 µm^2^ were automatically excluded from the measurements. Photographs of whole sections stained for ALP and vWF were taken with an Aperio scanner (Scanscope-XT, Aperio technologies, Vista, USA) using 40x objective. Analyses of the percentage myocardium, excluding endocardium, positive for each marker were done with a computerized morphometry system (Leica Qwin V3). Photographs of the fluorescent stainings were performed on a Leica SP5 system microscope (Leitz Wetzlar, Heidelberg, Germany); they were collected individually in the blue and red channels and merged thereafter. An average of 5 photographs were taken around the left ventricle and the pericyte coverage of microvessels was determined by counting NG2^+^/CD31^+^ vessels using a Image J computer analysis program.

### Gated SPECT/CT

Gated single photon emission computed tomography (gSPECT) acquisitions were made with the dedicated small-animal NanoSPECT/CT (Bioscan Europe, Ltd., Paris, France). Animals were anesthetized with Hypnorm (Fentanyl 0.26 mg/kg/Fluanisone 8.33 mg/kg, VetaPharma, Ltd., Leeds, UK) and Dormicum (Midazolam, 4.17 mg/kg, Roche, Woerden, the Netherlands) via intraperitoneal (i.p.) injection (1:2:1 Hypnorm:H_2_O:Dormicum; 120 µl/mouse). Serum Albumin (HSA) (Vasculosis, IBA Molecular, Gif-sur-Yvette, France) was labeled with 1–1.5 ml ^99m^Tc-pertechnetate. The radiotracer (150 µl) was injected intravenously (i.v.), with a total activity of about 50 MBq per mouse. Three-lead electrodes (3M red Dot 2282E, 3M, St.Paul, USA) were attached to both hind paws and right front paw of the mouse, placed on the animal bed in the prone position and connected to the integrated electrocardiography (ECG) monitor to measure heart rate (HR). Once a stable HR was established, a short X-ray topogram was made to set the field of view (FOV) and so focus on the thorax to reduce scan time. After the FOV was set, gated SPECT acquisition was started using a quadruple-head gamma camera high precision gantry, equipped with 4 pyramid collimators and 9 pinhole apertures (diameter 1.2 mm). The axial FOV was 16 mm. A 20% window centered on the 140 keV photoelectric peak of ^99m^Tc was used to acquire 20 projections with uniform angular sampling over a 360° radius into a 128×128 matrix Human X-ray topogram and SPECT acquisition were initiated directly after tracer administration. ECG-gated data were recorded in 8 time-bins per cardiac cycle. HiSPECT NG software (InVivoScope, Bioscan) was used to perform iterative reconstruction into 3D-datasets. Quantitative analysis of the reconstructed datasets was performed on a clinical e.soft (*syngo*-based) workstation (Siemens Medical Solutions, Siemens AG, Erlangen, Germany), using algorithms to automatically reconstruct a count based 3D model of the dimensions of the left ventricular (LV) end diastolic and systolic volumes (EDV, ESV). The ejection fraction (EF) was calculated based on the difference between EDV and ESV divided by EDV.

### Statistics

Data are expressed as mean ± SEM and groups were compared using non-parametric Mann–Whitney exact U-tests. Group differences were considered statistically significant at p<0.05. Statistical analyses were performed using SPSS version 20.

## Results

### Mouse health

There were no significant differences between groups in mean body weight or health, based on genotype or treatment or time-point (Table S2), except a small decrease in heart/bodyweight ratio in irradiated Eng^+/−^ mice after 20 weeks compared to age matched controls. There was no obvious telangiectasia in external organs (ears, paws) of unirradiated Eng+/− mice at termination of the experiment (maximum follow-up 40 weeks, mice aged 1 year).

### Impaired inflammatory response in irradiated hearts of Eng^+/−^ mice at 4 weeks

In order to identify genes and pathways potentially involved in the cardiac response to irradiation in Eng^+/−^ mice versus Eng^+/+^ mice, microarray and pathway analyses were performed using the software program IPA (a full list of gene expression levels after cardiac irradiation of Eng^+/−^ and Eng^+/+^ mice can be found at https://www.ebi.ac.uk/arrayexpress/experiments/E-MTAB-1488/. Known ingenuity functional and/or canonical pathway analysis was used to identify over-representation of radiation-correlated genes within known functional assignments (such as inflammatory response) and to generate hypotheses.

**Figure 1:**
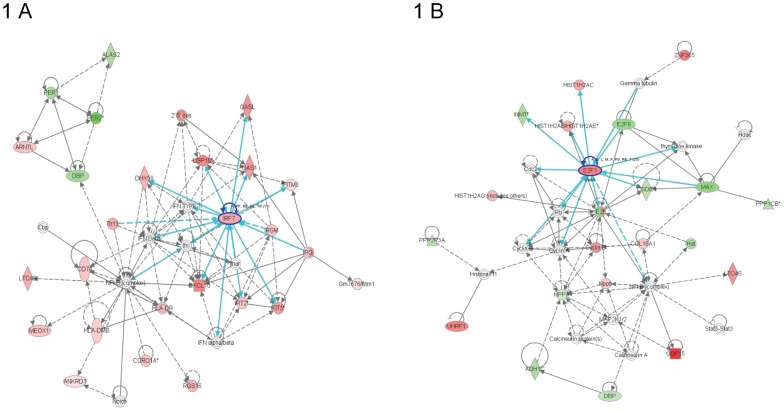
Graphical representation of the top networks of differentially regulated genes (4 weeks). Each network symbolizes the biological functions and/or diseases that were most significantly regulated 4 weeks after cardiac irradiation of Eng ^+/+^ mice (n = 5) (A) and Eng ^+/−^ mice (n = 4) (B). The genes marked in red represent the upregulated genes and in green the downregulated genes. The solid arrows represent direct interactions and the dotted arrows indirect interactions. Genes circled in dark blue represents central molecules and the light blue lines indicate interaction with other genes.

The most significantly altered network for Eng^+/+^ mice at 4 weeks after 16 Gy was classed as “behavior/ nervous system development and function”. Immune response regulating genes interferon regulatory factor 7 (IRF7) and chemokine (C-X-C motif) ligand 10 (CXCL10) were identified as central molecules and were significantly upregulated within this top network (Figure 1A). “Inflammatory response” and “immunological disease” were the top functional pathways significantly regulated 4 weeks after cardiac irradiation of Eng^+/+^ mice. This included 47 of the 115 molecules in these analyses (Table 1). The top upregulated genes were sarcolipin (SLN), myosin light chain 7 (MYL7) and myosin light chain 4 (MYL4), all of which are involved in maintaining cardiac contractility and function. Heat shock protein 70 (HSP70), involved in cardiomyocyte protection, was one of the top significantly down regulated genes (Table 2). In contrast, the most significantly altered network for Eng^+/−^ mice at 4 weeks after 16 Gy was classed as “hematological system development and function”. Cell proliferation and cell death-regulating gene E2 transcription factor was the central molecule within this top network (Figure 1B).

**Table 1:** Representation of the top network and functional pathways using IPA approach.

	Eng^+/+^ 16 Gy	Eng^+/−^ 16 Gy	Eng^+/+^ 16 Gy	Eng^+/−^ 16 Gy
	Network	Network	Functional pathway	Functional pathway
4 weeks	Nerv.system devlp. & funct. (51)	Hematl. system devlp.& funct. (36)	1. Inflammatory response	1. Cell-to-cell signlg.& inter.
			2. Immunological disease	2. Cell death
20 weeks	Inflammatory response (49)	Inflammatory response (40)	1. Inflammatory response	1. Cancer
			2. Cell-to-cell signlg.& inter.	2. Cardio.system devlp.& funct.
40 weeks	Lipid metabl, Molecl. Transp. (49)	Cellular movement (36)	1. Cancer	1. Tissue development
			2. Genetic disorder	2. Antigen presentation

Top networks for 4, 20 and 40 weeks after 16 Gy irradiation of Eng^+/+^ and Eng^+/−^ mice. Numbers in brackets represent the network score, which is explained in material and methods. The first two functional pathways for 4, 20 and 40 weeks after 16 Gy irradiation of Eng^+/+^ and Eng^+/−^ mice are also shown. Nerv.system devlp. & funct. : Nervous system development and function; Lipid metabl, Molecl. Transp.: Lipid metabolism, Molecular transport; Hematl. system devlp.& funct.: Hematological system development and function; Cell-to-cell signlg.& inter.: Cell-to-cell signaling and interaction; Cardio.system devlp.& funct.: Cardiovascular system development and function.

**Table 2:** Representation of differently regulated genes after 16 Gy irradiation using IPA approach.

	Eng^+/+^ 16 Gy	Eng^+/−^ 16 Gy	Eng^+/+^ 16 Gy	Eng^+/−^ 16 Gy
	Top upregulated genes	Top upregulated genes	Top downregulated genes	Top downregulated genes
4 weeks	SLN (6.2)	GDF15 (4.7)	C1orf51 (−3.0)	Hamp/Hamp2 (−4.9)
	MYL7 (5.6)	Hsp70 (3.4)	PER2 (−2.3)	CA3 (−3.3)
	MYL4 (4.8)	MKI67 (3.2)	CA4 (−2.2)	TGFbRIII (−3.3)
	GDF15 (4.4)	PBK (3.1)	Hsp70 (−1.9)	Kcnip2 (−3.3)
	Hamp/Hamp2 (4.3)	IRF7 (3.1)	COQ10B (−1.8)	MYL7 (−3.2)
20 weeks	CA3 (4.8)	IGHA (3.7)	CHTOP (−2.1)	CDO1 (−3.5)
	CFD (4.7)	GDF15 (3.5)	CXCL14 (−2.1)	Klra4 (−3.3)
	HP (3.4)	ESM1 (2.9)	Klra4 (−2.1)	Klk1b1 (−3.3)
	Hamp/Hamp2 (3.3)	HIST1H2AB (2.7)	ADH1C (−2.0)	INMT (−3.2)
	IRF7 (3.2)	PACSIN1 (2.6)	ALDOB (−1.9)	GUCY (−2.9)
40 weeks	ESM1 (3.9)	SLN (6.2)	Hsp70 (−4.3)	CCL21 (−5.0)
	SRGN (3.5)	MYL7 (5.7)	CXCL14 (−3.3)	INMT (−4.3)
	HPRT1 (3.3)	CLASP1 (4.3)	ADH1C (−3.0)	Mup1 (−4.3)
	HOXB7 (3.2)	DKK3 (3.9)	TDRD3 (−2.8)	ADH1C (−4.2)
	C1orf52 (3.2)	SORBS1 (3.9)	CPXM1 (−2.8)	Ifitm1 (−3.7)

The top 5 upregulated and downregulated genes per genotype are shown after 16 Gy at 4 weeks, 20 weeks and 40 weeks. Numbers in brackets show log_2_ ratio of sham treated mice versus 16 Gy irradiated mice.

**Figure 2:**
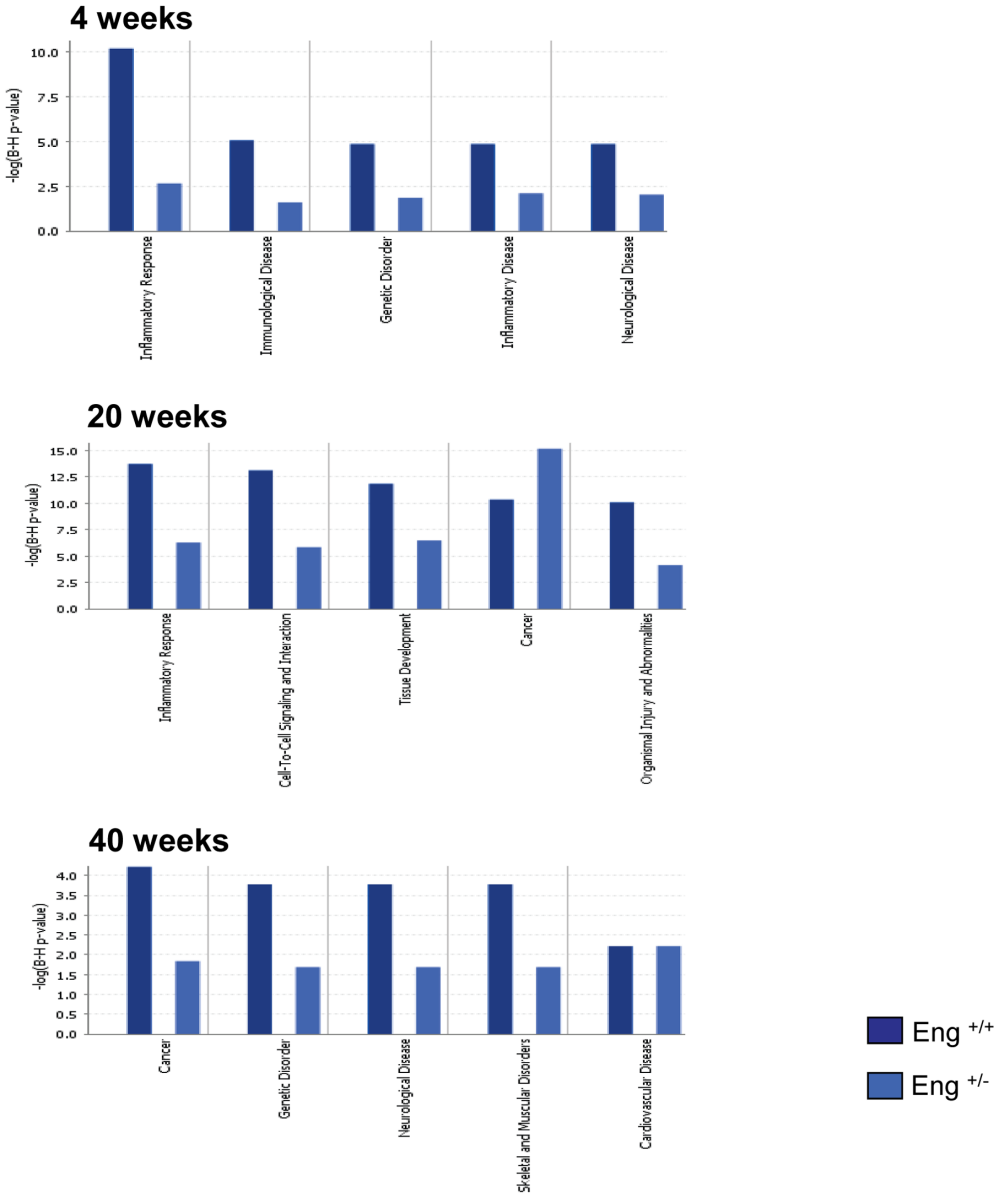
Comparison of top 5 functional pathways from Eng^+/+^ (dark blue) and Eng^+/−^ (light blue), generated by IPA analysis. Bars indicate top networks expressed and y-axis displays the – (log) significance. Taller bars are more significant than shorter bars. P-value display Benjamini-Hochberg multiple testing correction.

Inflammatory responses and immunological disease were much less prominent in Eng^+/−^ mice than Eng^+/+^ mice at 4 weeks after 16 Gy (Figure 2). Other upregulated functional pathways in Eng^+/−^ mice were “cell-to-cell signaling” and “cell death related pathways” (Table 1). These pathways include significant downregulation of genes related to binding of connective tissue cells and fibroblasts and significant upregulation of genes related to apoptosis of endothelial cells. Interestingly, cardiac function maintaining genes and cardiomyocyte protective genes (SLN, MYL7, MYL4, and HSP70) were oppositely regulated in these two mouse strains (Table 2).

**Figure 3:**
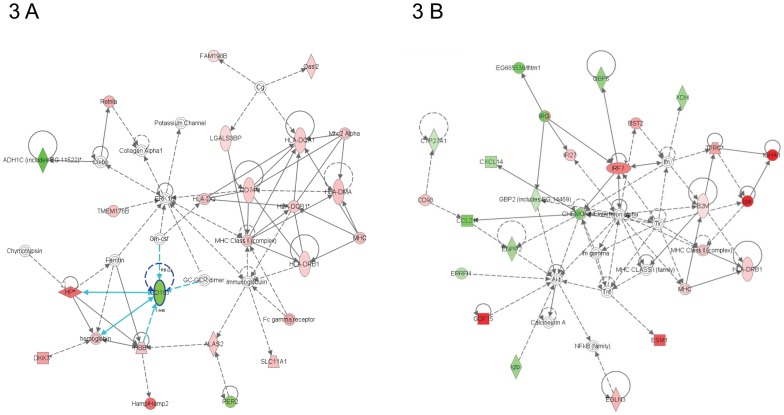
Graphical representation of the top network of differentially regulated genes (20 weeks). Each network symbolizes the biological functions and/or diseases that were most significantly regulated 20 weeks after cardiac irradiation of Eng ^+/+^ mice (n = 4–5) (A) and Eng ^+/−^ mice (n = 5) (B). The genes marked in red represent the upregulated genes and in green the downregulated genes. The solid arrows represent direct interactions and the dotted arrows indirect interactions. Genes circled in dark blue represents central molecules and the light blue lines direct interaction with other genes.

The most significantly altered network for Eng^+/+^ mice at 20 weeks after 16 Gy was inflammatory response (Figure 2). Acute phase-regulated receptor and signal-inducing macrophage protein CD163 (downregulated) was one of many central molecules within this network (Figure 3A). As at 4 weeks, Eng^+/+^ mice still showed significant upregulation in inflammatory response, with 55/142 genes in this functional pathway analysis (Table 1). Both complement immune system related gene (CFD) and carbonic anhydrase III (CA3), which are related to cardiac myocyte damage, were significantly upregulated top genes (Table 2). Analysis of Eng^+/−^ hearts at 20 weeks after 16 Gy also indicated inflammatory response as the top network. However, the score of this network was lower than in Eng^+/+^ mice (Figure 2) and inflammation did not emerge as one of the top functional pathways (Table 1). Central molecules within this network were interferon regulatory factor 7 (IRF7), beta-2-microglobulin (B2M) and CHEMOKINE complex (Figure 3B). Top upregulated genes indicated endothelial disorder (endothelial cell-specific molecule 1 (ESM1)) (Table 2).

**Figure 4:**
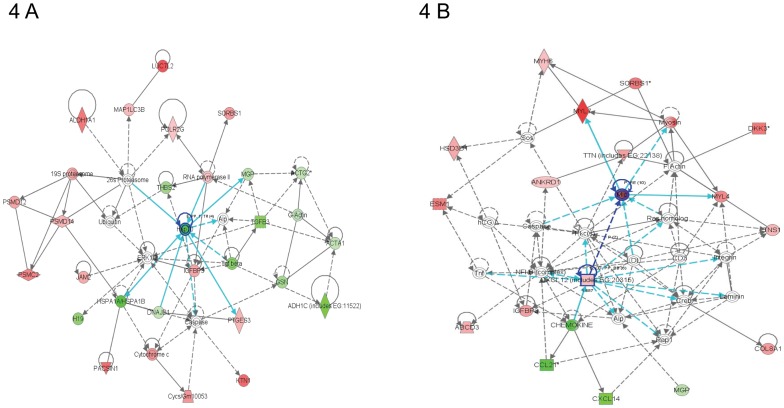
Graphical representation of the top network of differentially regulated genes (40 weeks). Each network symbolizes the biological functions and/or diseases that were most significantly regulated 40 weeks after cardiac irradiation of Eng ^+/+^ mice (n = 4–5) (A) and Eng ^+/−^ mice (n = 4–7) (B). The genes marked in red represent the upregulated genes and in green the downregulated genes. The solid arrows represent direct interactions and the dotted arrows indirect interactions. Genes circled in dark blue represents central molecules and the light blue lines direct interaction with other genes.

The most significantly altered network for Eng^+/+^ mice at 40 weeks after 16 Gy was “lipid metabolism and molecular transport”, with cardiomyocyte protective gene HSP70 as the central molecule (Figure 4A). HSP70 was also one of the top downregulated genes, indicating a lack of cardiomyocyte protection (Table 2). “Neurological disorder” and “cardiovascular disease” were two of the top functional pathways (Figure 2), both containing adrenergic, beta-1, receptor (ADRB1). ADRB1, which stimulates smooth muscle contraction and promotes increased contractility and heart rate, was significantly upregulated (Table 1). Inflammatory response was no longer detected at 40 weeks after irradiation. Top network for Eng^+/−^ mice at 40 weeks after 16 Gy was “cellular movement”. Two central molecules were involved within this network; myosin light chain (MLC) and chemokine (C-X-C motif) ligand 12 (CXCL12), and both were significantly upregulated (Figure 4B). Furthermore, many cardiac contractile stimulating genes were involved in this network (MYL7, MYL4, MYH6, tensin-1, titin and myosin). Again, the top upregulated genes were involved in cardiac contractility (SLN, MYL7) (Table 2). Inflammatory response and immunological disease were not within the top functional pathways at 40 weeks after 16 Gy but neurological disease, muscle disorder and cardiovascular disease were (Figure 2). Neurological disease and muscle disorder were more pronounced in Eng^+/+^ mice than to Eng^+/−^ mice. Cardiovascular disease was equally upregulated within both strains (Figure 2).

### Pro-fibrotic genes upregulated in Eng^+/+^ mice only at 4 weeks

**Figure 5:**
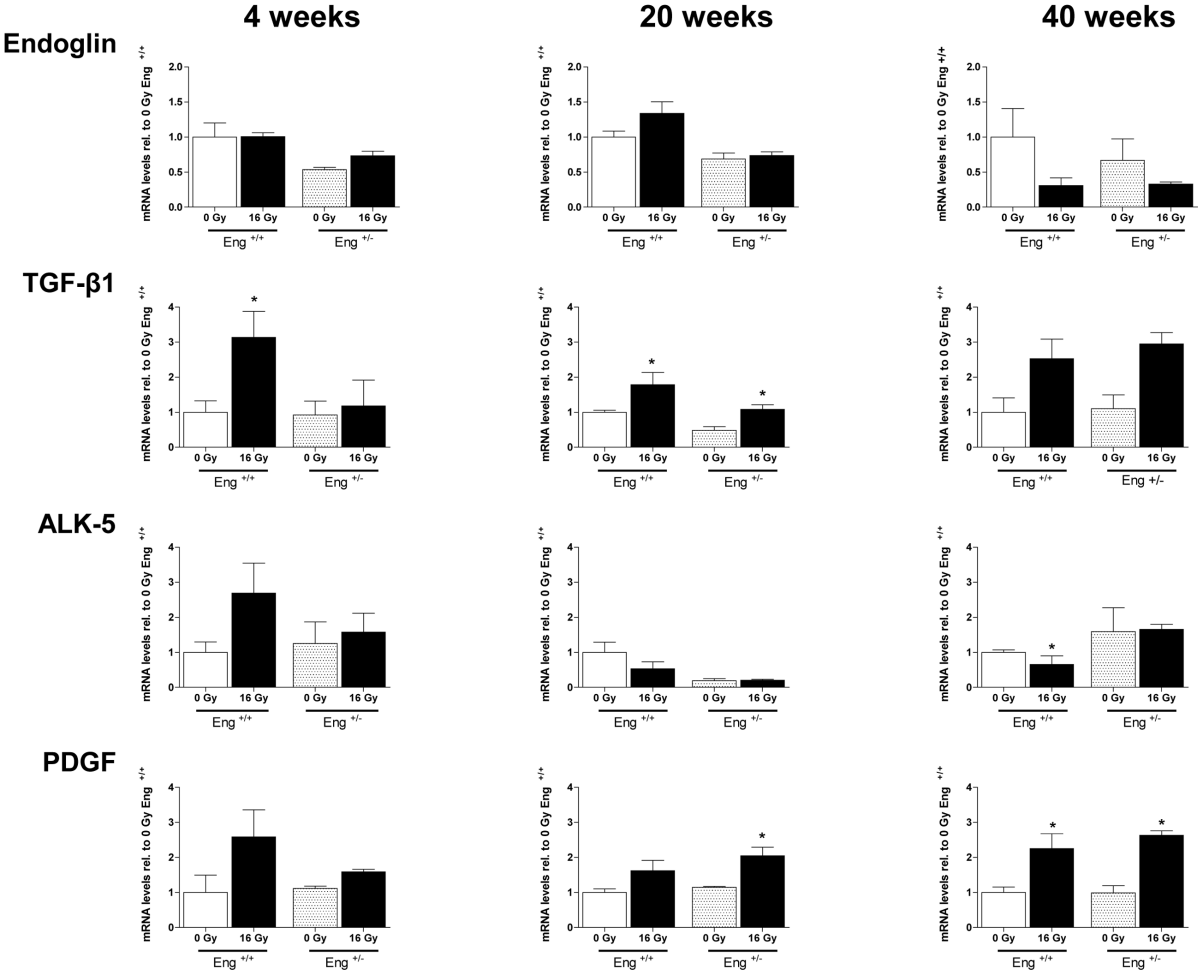
Expression of genes involved in TGFβ pathway measured by RT PCR. Each bar represents the average expression per group ± SEM. Values of sham-treated animals were set to 1. The graph show the fold-change in gene expression in irradiated mice relative to respective controls at 4 (n = 4–5), 20 (n = 4–5), and 40 (n = 4–7) weeks after 16 Gy irradiation.

Unirradiated Eng^+/−^ mice had reduced endoglin mRNA expression compared to Eng^+/+^ mice, as expected (Figure 5, top panels). Immunohistochemistry analysis confirmed that endoglin protein levels in hearts of Eng^+/−^ mice were also approximately half that of Eng^+/+^ mice (data not shown). By 40 weeks after irradiation, the mRNA levels of endoglin were decreased to <50% of control values in both strains, although this did not reach statistical significance. TGF-β1 was significant increased by 4 weeks after irradiation in Eng^+/+^ mice and in both strains at 20 weeks and 40 weeks (not significant) after irradiation. Irradiation did not alter the mRNA expression of fibrogenesis activator ALK5 in Eng^+/−^ mice but there was a non-significant increase in ALK5 in Eng^+/+^ mice at 4 weeks followed by significant decreased at 40 weeks after irradiation. PDGF showed a trend to increase in Eng^+/+^ mice at early times (4 weeks), with increases in both strains at later times (Figure 5). Profibrotic CTGF and PAI-1 were not altered significantly by radiation in either strain at 4, 20 or 40 weeks after irradiation (data not shown).

### Microvascular damage in Eng^+/+^ and Eng^+/−^ mice

After demonstrating significant differences between Eng^+/+^ mice and Eng^+/−^ mice in inflammatory, fibrogenic and survival pathway signaling in response to cardiac irradiation, we investigated this in more detail with respect to tissue morphology and function.

**Figure 6:**
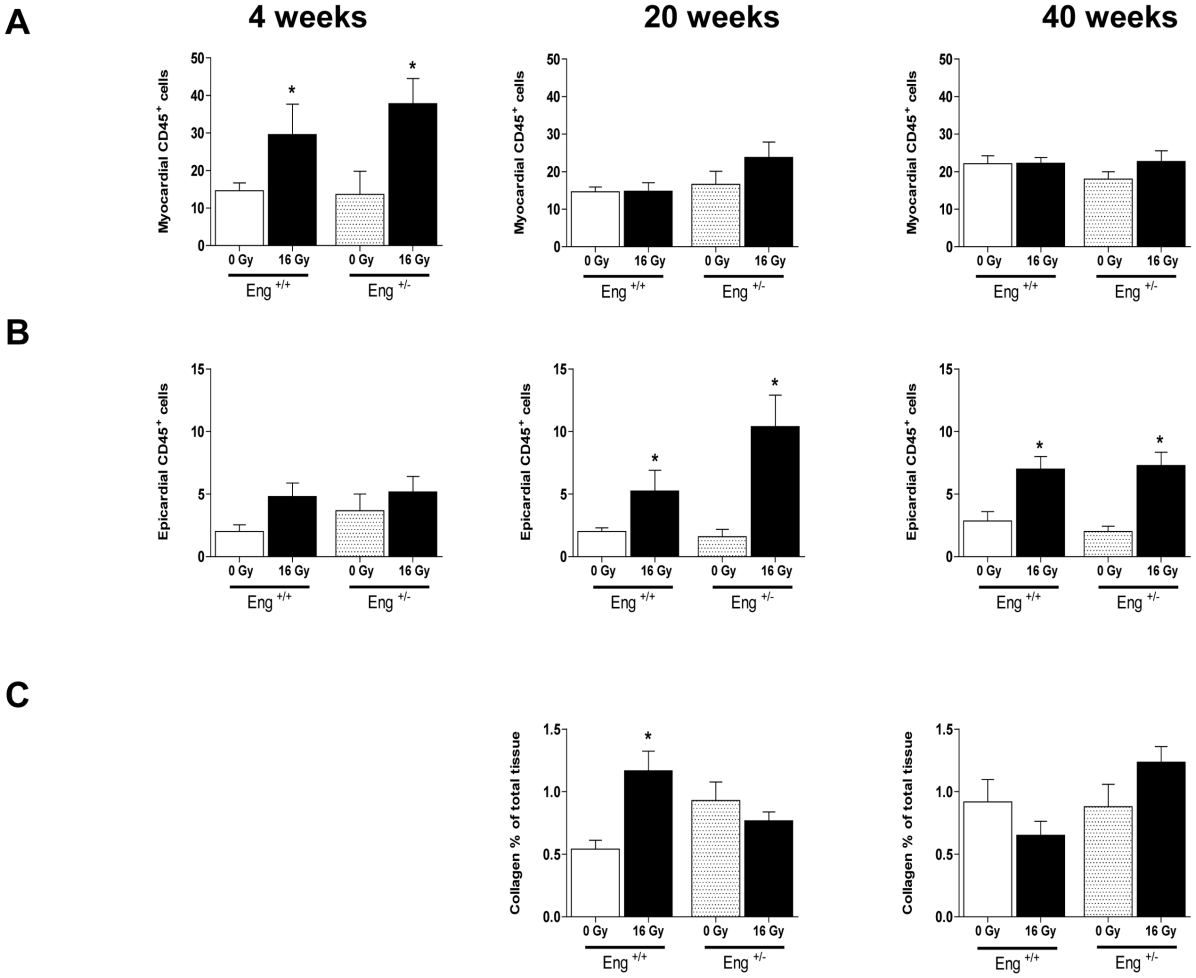
Inflammatory and fibrotic changes at 4, 20 and 40 weeks after irradiation or sham treatment. (A) Quantification of CD45+ cells per section in the myocardium and (B) epicardium. (C) Percentage interstitial collagen content of irradiated heart sections, relative to age-matched unirradiated controls. Values represent mean ± SEM with 3–6 mice in the 4 weeks group, 4–5 mice in the 20 weeks group and 5–7 in the 40 weeks group, *p<0.05 compared to age-matched unirradiated controls.

Epicardial thickness and myocardial thickness were not altered at any time-point after irradiation in either strain (data not shown). Irradiation led to a transient increase in CD45+ cells in the myocardium of both strains at 4 weeks and in the epicardium at 20 and 40 weeks. However, there were no differences in the inflammatory response noted between strains at the tissue level (Figure 6 A-B). Iron-containing macrophages, as an indicator of hemorrhage, were significantly increased in both myocardium and epicardium at 20 and 40 weeks after irradiation in both strains (data not shown). Collagen deposition in the myocardium was increased at 20 weeks after irradiation in Eng^+/+^ mice only, with no significant changes at 40 weeks in either strain (Figure 6 C).

**Figure 7:**
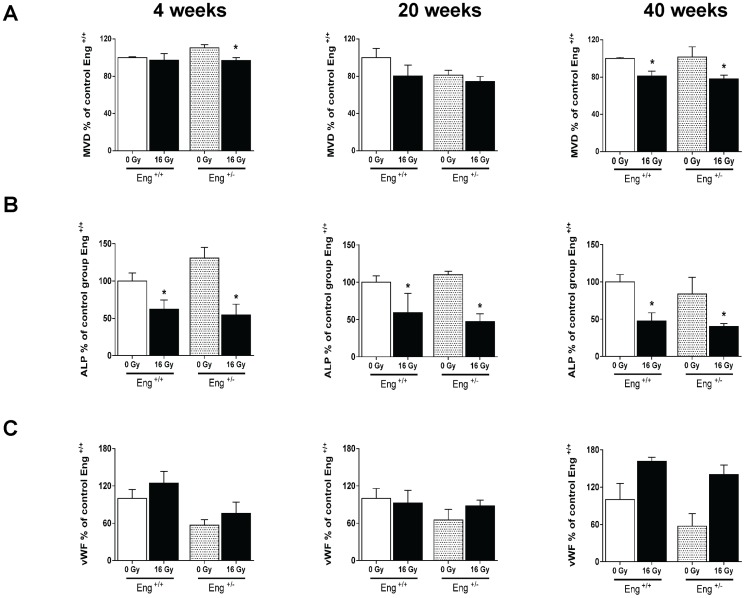
Microvascular alterations at 4, 20 or 40 weeks after irradiation or sham treatment. (A) MVD per unit area expressed as percentage of age matched unirradiated control values. (B) ALP positive tissue areas as % of age-matched unirradiated controls. (C) vWF positive tissue areas as % of age-matched unirradiated controls. Values represents mean ± SEM with 4–5 mice in the 4 and 20 weeks group and 4–7 mice in the 40 weeks group,*p<0.05 compared to age-matched unirradiated controls.

**Figure 8:**
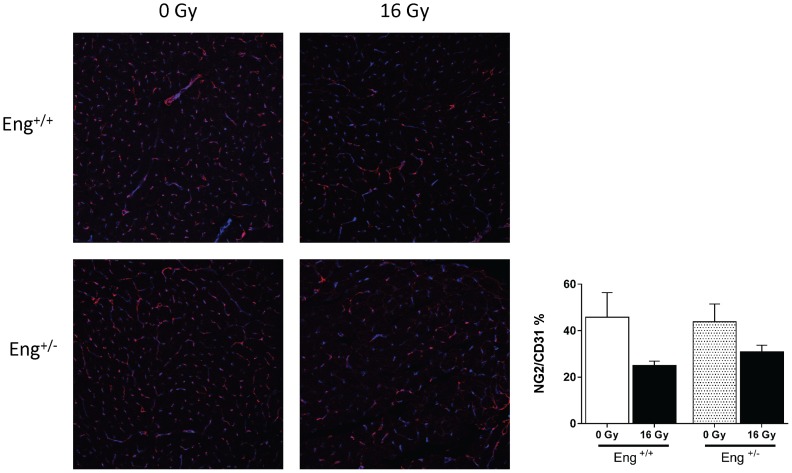
Pericyte coverage altered at 40 weeks after irradiation. Confocal imaging showing the effects of radiation on pericyte coverage (marked in red) on endothelial cells of the cardiac microvasculature (marked in blue). Graph displaying NG2/CD31 ratio as % of each individual treatment group and genotype. Values represents mean ± SEM with 4–5 mice in the 4 and 20 weeks group and 4–7 mice in the 40 weeks group,*p<0.05 compared to age-matched, unirradiated controls.

Microvascular density (MVD) decreased significantly at 4 weeks after irradiation in Eng^+/−^ mice and in both strains 40 weeks after irradiation (Figure 7 A). This was accompanied by endothelial damage, as shown by a marked decrease in ALP activity at 4, 20 and 40 weeks after irradiation, and increased expression of the thrombotic endothelial marker vWF (not significant) at 40 weeks in both strains (Figure 7 B–C). Microvascular stability, assessed by pericyte coverage, was decreased in irradiated mice at 40 weeks (not significant) but there were no differences between strains (Figure 8).

Since Eng+/− mice are known to be susceptible to microvascular defects as they age [13], [14], we also compared the microvascular density and functionality (pericyte coverage, ALP and vWF expression) in unirradiated Eng+/− and Eng+/+ mice at 40 weeks follow-up. There were no significant differences in any of these parameters between the strains (Figures 7, 8).

### Normalized cardiac function in Eng^+/−^ mice at 40 weeks

**Figure 9:**
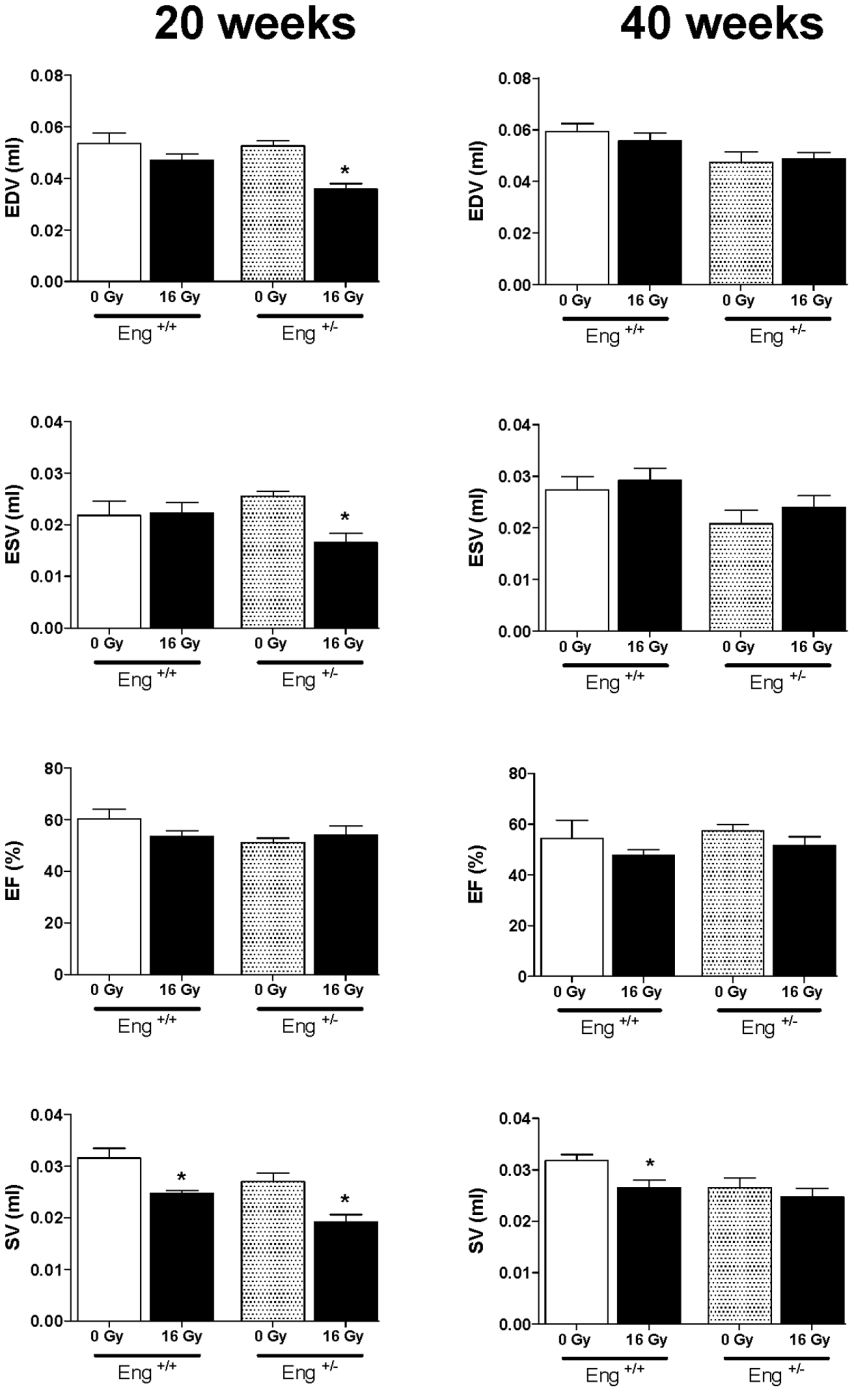
EDV, ESV, EF and SV measured by gated SPECT at 20 weeks or 40 weeks after irradiation or sham treatment. Values represent mean ± SEM (7–14 mice in each irradiated group), *p<0.05 compared to age-matched, unirradiated controls.

Cardiac function, evaluated by gated SPECT (^99m^Tc-HSA) showed modest decreases in EDV and ESV at 20 weeks after irradiation of Eng^+/−^ mice only. SV decreased in both strains with no changes in EF (Figure 9). Almost all cardiac function parameters had normalized to control levels at 40 weeks after irradiation.

## Discussion

Microvascular damage has been identified as a contributing factor in developing heart failure after high dose ionizing radiation [1], [15], [16], [17]. Endothelial cells are highly sensitive to radiation and aberrant signaling by damaged cells affects the pathological progression of radiation-induced tissue damage [18], [19], [20]. Endoglin is a co-receptor for TGF-β1; it is essential for angiogenesis and predominantly expressed in activated vascular endothelial cells [10], [11], [12], [13], [14], [21], [22]. In vivo studies have demonstrated that a mutation or deficit in endoglin causes microvascular damage. Mice deficient in endoglin develop vascular and cardiovascular damage, leading to death of Eng^−/−^ embryos or to the HHT phenotype in Eng^+/−^ mice, including endothelial cell degeneration, defects in endothelial junctions and incomplete smooth muscle cell coating of the vessel [13], [14], [21], [22]. Several studies have shown that endoglin is upregulated in an inflammatory environment and plays a role in lymphocyte trafficking and migration. Furthermore, endoglin haplo-insufficient mice have been shown to have a reduced inflammatory response and limited cardiac fibrosis after inflammatory stimuli [23], [24].

In this study gene expression analysis of irradiated hearts demonstrated a decreased inflammatory response in Eng^+/−^ mice compared to Eng^+/+^ mice. This was particularly evident during the early phase of radiation-induced normal tissue damage (4 weeks after 16 Gy). Genes involved in both inflammatory response and immunological related disease were less prominently regulated by radiation in Eng^+/−^ mice than in Eng^+/+^ mice. Similar results were previously obtained by Scharpfenecker et al. [25] in irradiated kidneys of Eng^+/+^ mice and Eng^+/−^ mice. In these studies the reduced endoglin levels in Eng^+/−^ mice were associated with both reduced expression of inflammatory cytokines (Ccr2, IL1b, IL6) and reduced inflammatory infiltration (CD45+ cells) in irradiated kidney [26]. In our study the reduced inflammatory response indicated from gene expression analysis was not reflected in reduced inflammatory infiltration in irradiated heart tissue.

Radiation-induced cardiac inflammation often precedes a late fibrotic response. In our study, fibrosis related genes TGF-β1, and to a lesser extent ALK5 and PDGF, were only upregulated in Eng^+/+^ mice during the early phase of radiation induced cardiac damage (4 weeks). Moreover, at 20 weeks after irradiation, immunohistochemical analysis showed significant collagen deposition in Eng^+/+^ mice only and not in Eng^+/−^ mice. Again, this is consistent with previous studies, in which endoglin haplo-insufficiency reduced fibrosis in irradiated kidneys and in a model of ischemic heart damage [23], [26].

With increased follow-up, our results indicated a shift away from the inflammatory response, although profibrotic genes TGF-β1 and PDGF were upregulated in both strains at 20 and 40 weeks follow up. This suggests that endoglin haplo-insufficiency only limits inflammation and fibrosis in the early phase of radiation-induced cardiac damage.

We had previously demonstrated that cardiac irradiation of wildtype C57BL/6 mice induced endothelial cell damage [3]. Since endoglin is a co-receptor of TGF-β1, and plays an important role in vascular morphogenesis, endothelial cell function and differentiation of pericytes and smooth muscle cells, one might expect increased endothelial cell damage and loosening of endothelial-pericyte interaction after irradiation in endoglin haplo-insufficient mice [13], [14], [27]. Our results did indicate an early reduction of MVD in irradiated hearts of Eng^+/−^ mice but functional damage in remaining vessels, ALP, vWF expression and pericyte coverage, was not greater in Eng^+/−^ than Eng^+/+^ mice.

Changes in cardiac function were modest and non-progressive but significant decreases in ESV and EDV were seen at 20 weeks in endoglin haplo-insufficient mice and decreased SV in both strains. Upregulation in genes involved in cardiac contractility (top network 16 Gy 40 weeks) in irradiated Eng^+/−^ hearts may have contributed to a normalization of cardiac function at 40 weeks after irradiation.

In summary, high dose radiation induced endothelial cell damage in cardiac microvasculature, which progressed in time. This microvascular tissue damage was independent of endoglin expression levels. However, our data do demonstrate that endoglin haplo-insufficiency limited the early inflammatory response and fibrosis in our radiation-induced mouse model of cardiac damage. Another model of acute pressure overload heart failure [23] showed that reduced endoglin expression had a more pronounced effect on cardiac outcome, with attenuated fibrosis preserved left ventricular function and improved survival. However, radiation-induced heart damage does not result in acute or severe hypoxia but stimulates progressive endothelial dysfunction and capillary loss and leads to a delayed myocardial damage without severe hypoxia. The underlying mechanisms precipitating myocardial fibrosis and heart failure are therefore different and the importance of endoglin in these different pathologies probably varies.

## Supporting Information

Table S1 Primers used for quantitative real-time PCR.
(DOC)

Table S2 Body and organ weights of mice at sacrifice. * indicates significant differences between irradiated and age matched control groups (p<0.05; Mann-Whitney U-test).
(DOC)

## References

[1] Boerma M, Kruse JJ, van Loenen M, Klein HR, Bart CI, et al (2004) Increased deposition of von Willebrand factor in the rat heart after local ionizing irradiation. Strahlenther Onkol 180: 109–116. 10.1007/s00066-004-1138-0 14762664

[2] Lauk S, Trott KR (1990) Endothelial cell proliferation in the rat heart following local heart irradiation. Int J Radiat Biol 57: 1017–1030. 1970990 10.1080/09553009014551131

[3] Seemann I, Gabriels K, Visser NL, Hoving S, te Poele JA, et al (2012) Irradiation induced modest changes in murine cardiac function despite progressive structural damage to the myocardium and microvasculature. Radiother Oncol 103: 143–150. 22112779 10.1016/j.radonc.2011.10.011

[4] Andrianifahanana M, Wilkes MC, Repellin CE, Edens M, Kottom TJ, et al (2010) ERBB receptor activation is required for profibrotic responses to transforming growth factor beta. Cancer Res 70: 7421–7430. 20841477 10.1158/0008-5472.CAN-10-0232 PMC3093933

[5] Martin M, Lefaix J, Delanian S (2000) TGF-beta1 and radiation fibrosis: a master switch and a specific therapeutic target? Int J Radiat Oncol Biol Phys 47: 277–290. 10802350 10.1016/s0360-3016(00)00435-1

[6] Anscher MS (2010) Targeting the TGF-beta1 pathway to prevent normal tissue injury after cancer therapy. Oncologist 15: 350–359. 20413640 10.1634/theoncologist.2009-S101 PMC3227962

[7] Barcellos-Hoff MH, Derynck R, Tsang ML, Weatherbee JA (1994) Transforming growth factor-beta activation in irradiated murine mammary gland. J Clin Invest 93: 892–899. 8113421 10.1172/JCI117045 PMC293960

[8] Scharpfenecker M, Kruse JJ, Sprong D, Russell NS, Ten Dijke P, et al (2009) Ionizing radiation shifts the PAI-1/ID-1 balance and activates notch signaling in endothelial cells. Int J Radiat Oncol Biol Phys 73: 506–513. 19147015 10.1016/j.ijrobp.2008.09.052

[9] van Meeteren LA, Goumans MJ, ten Dijke P (2011) TGF-beta receptor signaling pathways in angiogenesis; emerging targets for anti-angiogenesis therapy. Curr Pharm Biotechnol 12: 2108–2120. 21619534 10.2174/138920111798808338

[10] Gougos A, Letarte M (1988) Identification of a human endothelial cell antigen with monoclonal antibody 44G4 produced against a pre-B leukemic cell line. J Immunol 141: 1925–1933. 3262644

[11] Lebrin F, Goumans MJ, Jonker L, Carvalho RL, Valdimarsdottir G, et al (2004) Endoglin promotes endothelial cell proliferation and TGF-beta/ALK1 signal transduction. EMBO J 23: 4018–4028. 15385967 10.1038/sj.emboj.7600386 PMC524335

[12] Li C, Hampson IN, Hampson L, Kumar P, Bernabeu C, et al (2000) CD105 antagonizes the inhibitory signaling of transforming growth factor beta1 on human vascular endothelial cells. FASEB J 14: 55–64. 10627280 10.1096/fasebj.14.1.55

[13] Arthur HM, Ure J, Smith AJ, Renforth G, Wilson DI, et al (2000) Endoglin, an ancillary TGFbeta receptor, is required for extraembryonic angiogenesis and plays a key role in heart development. Dev Biol 217: 42–53. 10625534 10.1006/dbio.1999.9534

[14] Bourdeau A, Dumont DJ, Letarte M (1999) A murine model of hereditary hemorrhagic telangiectasia. J Clin Invest 104: 1343–1351. 10562296 10.1172/JCI8088 PMC409846

[15] Fajardo LF, Stewart JR (1971) Capillary injury preceding radiation-induced myocardial fibrosis. Radiology 101: 429–433. 5114783 10.1148/101.2.429

[16] Fajardo LF, Stewart JR (1973) Pathogenesis of radiation-induced myocardial fibrosis. Lab Invest 29: 244–257. 4724850

[17] Lauk S, Kiszel Z, Buschmann J, Trott KR (1985) Radiation-induced heart disease in rats. Int J Radiat Oncol Biol Phys 11: 801–808. 3980275 10.1016/0360-3016(85)90314-1

[18] Juncos LI, Cornejo JC, Gomes J, Baigorria S, Juncos LA (1997) Abnormal endothelium-dependent responses in early radiation nephropathy. Hypertension 30: 672–676. 9323003 10.1161/01.hyp.30.3.672

[19] Paris F, Fuks Z, Kang A, Capodieci P, Juan G, et al (2001) Endothelial apoptosis as the primary lesion initiating intestinal radiation damage in mice. Science 293: 293–297. 11452123 10.1126/science.1060191

[20] Ran XZ, Ran X, Zong ZW, Liu DQ, Xiang GM, et al (2010) Protective effect of atorvastatin on radiation-induced vascular endothelial cell injury in vitro. J Radiat Res 51: 527–533. 20921821 10.1269/jrr.09119

[21] Lopez-Novoa JM, Bernabeu C (2010) The physiological role of endoglin in the cardiovascular system. Am J Physiol Heart Circ Physiol 299: H959–974. 20656886 10.1152/ajpheart.01251.2009

[22] McAllister KA, Grogg KM, Johnson DW, Gallione CJ, Baldwin MA, et al (1994) Endoglin, a TGF-beta binding protein of endothelial cells, is the gene for hereditary haemorrhagic telangiectasia type 1. Nat Genet 8: 345–351. 7894484 10.1038/ng1294-345

[23] Kapur NK, Wilson S, Yunis AA, Qiao X, Mackey E, et al (2012) Reduced endoglin activity limits cardiac fibrosis and improves survival in heart failure. Circulation 125: 2728–2738. 22592898 10.1161/CIRCULATIONAHA.111.080002 PMC4774533

[24] Rossi E, Sanz-Rodriguez F, Eleno N, Duwell A, Blanco FJ, et al (2013) Endothelial endoglin is involved in inflammation: role in leukocyte adhesion and transmigration. Blood 121: 403–415. 23074273 10.1182/blood-2012-06-435347

[25] Scharpfenecker M, Floot B, Russell NS, Stewart FA (2012) The TGF-beta co-receptor endoglin regulates macrophage infiltration and cytokine production in the irradiated mouse kidney. Radiother Oncol 105: 313–320. 23022174 10.1016/j.radonc.2012.08.021

[26] Scharpfenecker M, Floot B, Russell NS, Ten Dijke P, Stewart FA (2009) Endoglin haploinsufficiency reduces radiation-induced fibrosis and telangiectasia formation in mouse kidneys. Radiother Oncol 92: 484–491. 19576647 10.1016/j.radonc.2009.06.013

[27] Li DY, Sorensen LK, Brooke BS, Urness LD, Davis EC, et al (1999) Defective angiogenesis in mice lacking endoglin. Science 284: 1534–1537. 10348742 10.1126/science.284.5419.1534

