# Supplementary material for: Endoglin Haplo-Insufficiency Modifies the Inflammatory Response in Irradiated Mouse Hearts without Affecting Structural and Mircovascular Changes
Source: PLoS One. 2013 Jul 24;8(7):e68922. doi: 10.1371/journal.pone.0068922 (PMC3722207; doi:10.1371/journal.pone.0068922)
Supplement: Table S1 — Primers used for quantitative real-time PCR. (DOC) [file pone.0068922.s001.doc]

| **Gene** | **Forward** | **Reverse** |
| --- | --- | --- |
| *Eng* | CTCCATGCGCCTGAACATC | GTGATACCCAGTACAGAGGGCAG |
| *Gapdh* | TCACCACCATGGAGAAGGC | GCTAAGCAGTTGGTGGTGCA |
| *Pdgfb* | GATCTCTCGGAACCTCATCG | GGCTTCTTTCGCACAATCTC |
| *Tgfb1* | TTGCTTCAGCTCCACAGAGA | TGGTTGTAGAGGGCAAGGAC |
| *Pai-1* | TCTTGCATCGCCTGCCAT | GGACCTTGAGATAGGACAGTGCTT |
| *Alk5* | CTGCCATAACCGCACTGTCA | AAATGAAAGGGCGATCTAGTGATG |
| *CTGF* | GGGCCTCTTCTGCGATTTC | ATCCAGGCAAGTGCATTGGTA |
| *Alk1* | GTGTGGCGCGGTTCGT | CTCATCTCGTGAGGAGAAAATCTTG |
| *ID1* | AGCCCTTCAGGAGGCAAGAG | GCGGTAGTGTCTTTCCCAGAGAT |

**Supporting information**

Table S1: Primers used for quantitative real-time PCR
