# Supplementary material for: Endoglin Haplo-Insufficiency Modifies the Inflammatory Response in Irradiated Mouse Hearts without Affecting Structural and Mircovascular Changes
Source: PLoS One. 2013 Jul 24;8(7):e68922. doi: 10.1371/journal.pone.0068922 (PMC3722207; doi:10.1371/journal.pone.0068922)
Supplement: Table S2 — Body and organ weights of mice at sacrifice. * indicates significant differences between irradiated and age matched control groups (p<0.05; Mann-Whitney U-test). (DOC) [file pone.0068922.s002.doc]

| **Treatment** | **Body weight (g)** | **Heart weight (g)** | **Lung weight (g)** | **Heart/body weight (g)** |
| --- | --- | --- | --- | --- |
| *4 weeks*  0 Gy Eng^+/+^  16 Gy Eng^+/+^  0 Gy Eng^+/-^  16 Gy Eng^+/-^ | 30.1 ± 2.0  29.5 ± 1.3  29.5 ± 2.5  28.8 ± 1.9 | 0.192 ± 0.02  0.189 ± 0.03  0.173 ± 0.03  0.175 ± 0.02 | 0.158 ± 0.01  0.156 ± 0.01  0.159 ± 0.02  0.155 ± 0.01 | 6.4 ± 0.7  6.4 ± 1.2  5.8 ± 0.9  6.1 ± 0.8 |
| *20 weeks*  0 Gy Eng^+/+^  16 Gy Eng^+/+^  0 Gy Eng^+/-^  16 Gy Eng^+/-^ | 34.4 ± 3.3  32.5 ± 1.9  30.1 ± 4.5  32.0 ± 2.0 | 0.192 ± 0.02  0.182 ± 0.02  0.168 ± 0.03  0.155 ± 0.02 | 0.168 ± 0.01  0.167 ± 0.01  0.153 ± 0.01  0.165 ± 0.01 | 5.6 ± 0.4  5.6 ± 0.5  5.7 ± 0.8  4.9 ± 0.6 ***** |
| *40 weeks*  0 Gy Eng^+/+^  16 Gy Eng^+/+^  0 Gy Eng^+/-^  16 Gy Eng^+/-^ | 34.0 ± 2.7  34.7 ± 5.8  35.2 ± 3.7  36.2 ± 4.9 | 0.196 ± 0.02  0.196 ± 0.02  0.188 ± 0.02  0.185 ± 0.02 | 0.186 ± 0.02  0.181 ± 0.02  0.176 ± 0.01  0.177 ± 0.01 | 5.8 ± 0.5  5.7 ± 0.7  5.4 ± 0.5  5.1 ± 0.5 |

**Supporting information**

Table S2: Body and organ weights of mice at sacrifice

* indicates significant differences between irradiated and age matched control groups

(p <0.05; Mann-Whitney U-test)
